# Evaluation of Cancellous Bone Density from C3 to L5 in 11 Body Donors: CT Versus Micro-CT Measurements

**DOI:** 10.3390/jcm14041059

**Published:** 2025-02-07

**Authors:** Guido Schröder, Estelle Akl, Justus Hillebrand, Andreas Götz, Thomas Mittlmeier, Steffi S. I. Falk, Laura Hiepe, Julian Ramin Andresen, Reimer Andresen, Dirk Flachsmeyer-Blank, Hans-Christof Schober, Änne Glass

**Affiliations:** 1 Clinic for Orthopaedics and Trauma Surgery, Sana Hospital Bad Doberan, Academic Teaching Hospital of the University of Rostock, 18209 Hohenfelde, Germany; dirk.flachsmeyer-blank@sana.de; 2 Institute for Diagnostic and Interventional Radiology, Pediatric and Neuroradiology, Rostock University Medical Center, 18057 Rostock, Germany; estelle.akl@med.uni-rostock.de (E.A.); justus.hillebrand@med.uni-rostock.de (J.H.); 3 Institute for Biomedical Technology, Rostock University Medical Center, Warnemünde, 18119 Rostock, Germany; andreas.goetz@med.uni-rostock.de; 4 Department of Trauma Surgery, Hand and Reconstructive Surgery, Rostock University Medical Center, 18057 Rostock, Germany; thomas.mittlmeier@med.uni-rostock.de (T.M.); steffi.falk@med.uni-rostock.de (S.S.I.F.); 5 Institute of Anatomy, Rostock University Medical Center, 18057 Rostock, Germany; laura.hiepe@med.uni-rostock.de; 6 Division of Trauma Surgery, Department of Orthopaedics and Trauma Surgery, Medical University of Vienna, Währinger Gürtel 18–20, 1090 Vienna, Austria; julian.andresen@meduniwien.ac.at; 7 Institute of Diagnostic and Interventional Radiology/Neuroradiology, Westküstenklinikum Heide, Academic Teaching Hospital of the Universities of Kiel, Lübeck and Hamburg, 25746 Heide, Germany; randresen@wkk-hei.de; 8 OrthoCoast, Medical Office of Orthopedics and Osteology, 17438 Wolgast, Germany; hcr.schober@gmx.de; 9 Institute for Biostatistics and Informatics in Medicine and Ageing Research, Rostock University Medical Center, 18057 Rostock, Germany; aenne.glass@uni-rostock.de

**Keywords:** Hounsfield units, spine, osteoporosis, bone density, CT morphology

## Abstract

**Introduction:** Comparative studies on Hounsfield units (HU) and bone volume fraction (BVF%) for the demonstration of cancellous bone density in the entire spine and in the various intravertebral regions are rare. The aim of the present study was to determine HU in various segments and sectional planes (sagittal, axial, coronary) of the spine and their description in the context of bone density measurement on micro-CT, as well as the significance of the values for bone loss and fracture risk. **Materials/Methods:** The spines of 11 body donors were analyzed by means of high-resolution spiral CT and micro-CT. Vertebral deformities were identified on sagittal reformations and classified. Cancellous bone density in the individual vertebrae from C3 to L5, expressed in HU, was measured on CT images (in all 242 vertebral bodies). For this purpose, a manually positioned ROI was established in mid-vertebral cancellous bone in the axial, sagittal, and coronary planes. Using a Jamshidi^®^ needle, we obtained 726 specimens from prepared vertebrae extracted from three quadrants (QI: right-sided edge, QII: central, QIII: left-sided edge) and analyzed these on a micro-CT device (SKYSCAN 1172, RJL Micro & Analytic GmbH, Germany). The study design with multiple measurements was reflected by a General Linear Model Repeated Measures. The model was adjusted to the bone density values of both procedures (HU, BVF%) in the viewed sectional planes and quadrants for 22 vertebrae, with the predictors gender and fracture status, controlled for age and body mass index (BMI). Analysis of variance provided estimations of density values and comparisons of several subgroups. **Results:** All spines were osteoporotic. Both procedures revealed a significant reduction in cancellous bone density from C3 to L5 (*p* ≤ 0.018). Gender (*p* = 0.002) and fracture status (*p* = 0.001) have an impact on bone density: men have higher bone density values than women; cases with fewer fractures also have higher bone density values. CT revealed both effects (*p* = 0.002 for each) with greater clarity. HU on CT measurements in the axial plane showed higher density values than in the sagittal or coronary planes. CT measurement profiles along the spine as well as along the individual profiles of the 11 body donors were independent of the measured quadrants, but the micro-CT measurements were not. **Discussion:** The craniocaudal reduction in bone density was demonstrated in different degrees of clarity by the two procedures. Likewise, the procedure-related visualization of differences in cancellous bone density between genders, fracture groups, sectional planes, and quadrants indicates the need for a better understanding of the advantages of each procedure for patient-oriented approaches to the diagnosis of osteoporosis. Future research should be focused on the determination of standard values and their clinical application for the prevention and treatment of osteoporosis.

## 1. Background

Osteoporosis (OP) is a systemic skeletal disease marked by reduced bone mass and weakening of the microarchitecture of bone [1]. The prevalence of OP in the European Union, the United Kingdom and Switzerland is 5.6% (22.1% in women, 6.6% in men) (SCOPE-Study) [2]. Thus, OP is a significant health problem, especially because of the associated high risk of pathological fractures. So-called major osteoporotic fractures, also known as fractures typically associated with osteoporosis, increase markedly after the age of 50 years in women and after the age of 60 years in men [3]. Thus, elderly persons are subject to a higher risk of osteoporotic fractures, which also lead to poor quality of life, disability, loss of independence, referral to care homes, and higher mortality rates [4]. A variety of radiographic modalities is used to assess the risk of fracture and bone quality. Diagnostic radiological investigations play a decisive role in the prevention and treatment of OP; Hounsfield units (HU) are used to an increasing extent as an indicator of cancellous bone density and fracture risk [5,6]. Micro-computed tomography (micro-CT) permits three-dimensional ex vivo assessment of bone morphology [7]. It is used to measure a number of variables, including bone volume fraction (BVF%). The trabecular microarchitecture of bone as well as variations in cancellous bone density in the various segments of the spine are important factors underlying the emergence of insufficiency fractures [8,9]. Nonetheless, studies analyzing HU values along the entire spine are rare [9]. We lack comprehensive investigations of cancellous bone density including a systematic documentation of density values appearing in the various segments and sectional planes of the spine (axial, sagittal, coronary). It would be interesting to obtain data about cancellous bone density (HU) and microarchitecture (BVF) in one and the same vertebral body and segment of the spine. The aim of the present study was to analyze HU values along the spine in various sectional planes and quadrants to compare them with the corresponding BVF values and thus to assess the potential relevance of HU measurements for the estimation of bone loss and fracture risk. Based on the analysis of HU values in the entire spine, the study was expected to provide new data about the distribution of bone density and factors influencing bone density. The investigation considered the regional variability in trabecular microarchitecture to better understand OP pathophysiology. The following research questions were addressed:

What are the similarities and differences between the various radiological investigation modalities?

Specifically, do systematic differences exist in bone density values in the individual sectional planes and selected quadrants? Do trends exist in the values of bone density over the 22 vertebrae from C3 to L5? What are the factors that influence the density of cancellous bone?

## 2. Materials and Methods

### 2.1. Study Design and Group Allocation

The present investigation was a single-arm cadaver study at the Rostock University Medical Center. This study is based methodologically on previously published own research, all according to the ethic approval code A2017-0072 [9,10,11,12].

### 2.2. Recruitment and Ethics

The participants of the study were persons who had enrolled in the body donor program at the Institute of Anatomy of Rostock University Medical Center during their lifetimes and had consented voluntarily to donate their bodies to scientific research after their death. Medical information about the body donors was limited. Primarily, we knew the cause of death. The methods used to obtain tissue were in accordance with the ethical standards of the Declaration of Helsinki; this study was reviewed and approved by the appropriate regional ethics committee for medical research of Rostock University (No. A 2017-0072).

### 2.3. Inclusion and Exclusion Criteria

The clinical investigation comprised cadavers from which we were able to extract complete spines with vertebral bodies from C3 to L5, free of anatomical deformities or severe bone diseases such as tumors or bone metastases. In addition, the body donors were expected to be of advanced age (above 65 years), and the spines were required to show no signs of growth retardation, Paget’s disease, spinal fusion, or the formation of block vertebrae. Likewise, body donors who had undergone surgery on the spine with the use of foreign material were excluded. The implementation of inclusion and exclusion criteria was executed by a multidisciplinary team comprising experienced anatomists and senior residents in the final stages of their orthopedic and trauma surgery specialization. This team conducted the spinal column extractions, employing their expertise to perform initial macroscopic assessments for anatomical anomalies. To ensure objective evaluation of anatomical deformities and severe osseous pathologies, standardized CT-imaging was performed on all specimens. The radiographs were subsequently analyzed using a double-blind approach by two independent board-certified radiologists, both with substantial research experience in osteology. This dual-reader methodology significantly enhanced the accuracy and reliability of identifying relevant spinal pathologies, minimizing potential observer bias. The multitiered assessment protocol, leveraging both hands-on anatomical expertise and advanced imaging analysis, facilitated a comprehensive and rigorous application of the predefined inclusion and exclusion criteria. The synergy between direct clinical examination by specialists and quantitative radiological assessment provided a holistic evaluation of each subject against the established parameters. All personnel involved in this process possessed requisite qualifications and extensive experience in their respective domains. The anatomists and orthopedic residents brought a deep understanding of musculoskeletal structures and pathologies, while the radiologists contributed specialized knowledge in bone imaging interpretation. This amalgamation of diverse expertise significantly bolstered the robustness and validity of the selection process, ensuring that only specimens meeting the stringent criteria were included in the subsequent analyses.

### 2.4. Extraction of Spines and Cancellous Bone

After the postmortal stage, the cadavers were perfused through the left-sided femoral artery with a 96% ethanol solution at a pressure of 0.5 bar. This was followed by preservation in a free-floating state, in a 0.5% aqueous phenol solution. The spines of the body donors were exposed and extracted in prone position. The prepared specimens were stored in a 70% ethanol solution at 4 °C for subsequent imaging and aspiration of cancellous bone. The aspiration of cancellous bone was performed from the ventral-dorsal aspect in the center, as well as in the marginal areas of the vertebrae. Precise positioning was ensured by controlling the same on CT in order to take any existing fusion fractures into account. In cases of fusion fractures, there was still adequate uncompromised cancellous tissue for the extraction of biopsy specimens. Using a Jamshidi^®^ needle (8 gauge, 3.263 mm), a total of 726 cancellous bone cylinders were obtained from 242 previously exposed vertebrae, from the ventromedial and marginal areas, and each prepared for further investigation in an Eppendorf reaction vessel (1.5 mL).

### 2.5. Diagnostic Imaging

#### 2.5.1. CT and QCT

**Figure 1:**
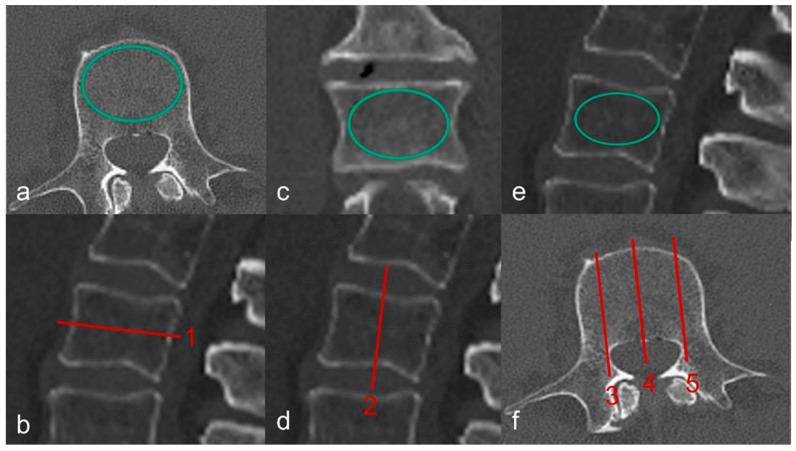
Determining Hounsfield units based on a mid-vertebral region of interest in the axial (**a**,**b**), coronary (**c**,**d**), and sagittal (**e**,**f**) plane. (**b**) shows the mid-vertebral height of the axial section (1). (**d**) displays the mid-vertebral height of the coronal section (2). (**f**) illustrates the mid-vertebral height of the sagittal section centrally (4), as well as laterally on the **right** (3) and **left** (5) sides.

In order to create realistic conditions for clinically accurate and anthropometric measurements, the extracted spines were submerged carefully in a PLEXIGLAS^®^ (PMMA) water phantom measuring 125 cm in length and 25 cm in diameter. Care was taken to ensure there were no air pockets. The donor spines were subjected to a high-resolution spiral CT investigation (GE Revolution EVO/64-slice CT/lateral scanogram) with an axial slice thickness of less than 1 mm, and with axial (Figure 1a,b), coronary (Figure 1c,d) and sagittal (Figure 1e,f) reformations with a slice thickness of 2 mm. Vertebral deformities were identified and graded on the sagittal reformations. The bone mineral density (BMD) of cancellous bone was determined on quantitative CT (GE Revolution EVO/64-slice computed tomography device and Mindways Software (version 4.2.3) 3D Volumetric QCT Spine, Austin, TX, USA). The measurement was performed on a volume block at the levels of L1, L2, and L3. The mean value—given in mg/cm^3^—was used to estimate the presence of OP.

#### 2.5.2. Micro-CT Images and Evaluation of Microarchitecture

**Figure 2:**
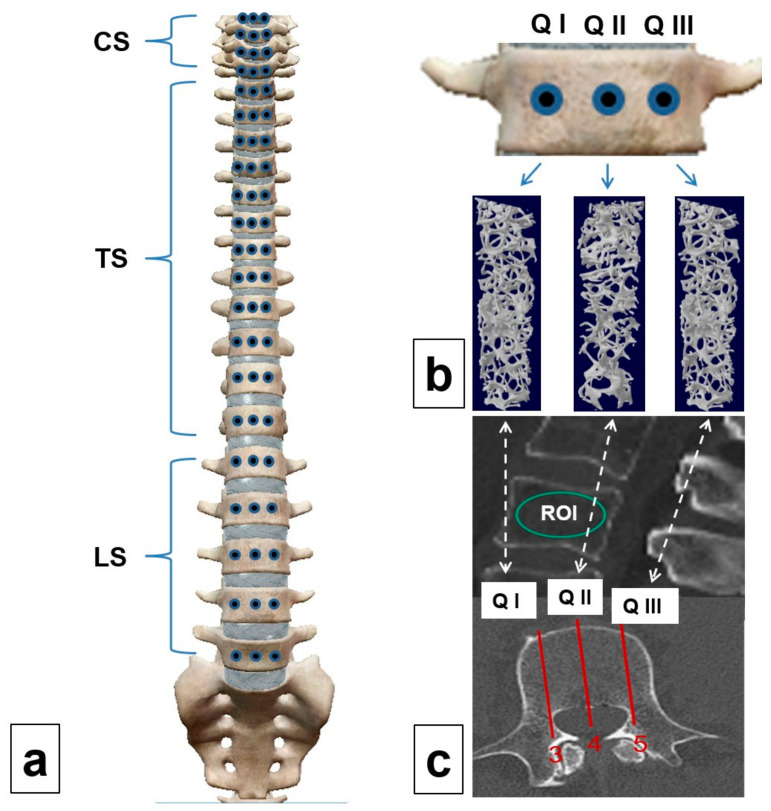
Schematic illustration of the extraction of cancellous bone cylinders (**a**) from the cervical (CS), thoracic (TS), and lumbar spine (LS); quadrant biopsies (**b**) from the marginal areas (QI and QIII) and the central quadrant (QII) for the determination of bone volume fraction (BVF) on micro-CT; determination of Hounsfield units using a region of interest in the sagittal (**c**) sectional plane in a central region (4) and in 2 marginal areas (3 and 5), which were deemed comparable to the biopsy quadrants on micro-CT. This was followed by a comparison of cancellous bone density determined on CT and micro-CT (QI or right-sided edge equals area 3, QII or the central region equals area 4, and Q III or the left-sided edge equals area 5).

The cancellous bone cylinders were obtained with the aid of micro-CT (SKYSCAN 1172, RJL Micro & Analytic Company, Karlsdorf Neuthart, Germany). For this purpose, we used flat-field correction and a comparison with phantoms (reference) at densities of 0.25 g/cm^3^ and 0.75 g/cm^3^. The settings for the scanning procedure were set as follows: filter AI 0.5, resolution 640 × 512 pixels, pixel size 19.9 µm, isotropic nominal voxel size 35 mm (field of view 70 mm, X-ray source 100 kV, 100 µA). The trabecular region of interest was defined manually in order to exclude the cortical component of the vertebra. Bone volume fraction (BVF, %) was the measured parameter of trabecular microarchitecture. A schematic illustration of the sampling procedure is shown in Figure 2.

### 2.6. Statistics

All descriptive statistics of quantitative characteristics (age, BMI, BMD) are shown as means ± standard deviation (SD). The study design with multiple measurements for the outcome of cancellous bone density was reflected by a General Linear Model Repeated Measures. The procedure provides an analysis of variance for several subgroups and factor levels. The model was fitted to the bone density values obtained from micro-CT (BVF%) and CT (HU) in the 3 sectional planes and measured quadrants QI, QII, QIII for 22 vertebrae using the binary predictors gender [female, male] and fracture status [≤1 fracture, >1 fracture], adjusted for covariates age [years] and BMI [kg/m^2^]. The assumption of normality was tested with the Shapiro–Wilk test. The resulting bone density values of investigated subgroups and factor levels are given as estimated marginal means ± SD, obtained from the model. Null hypotheses about the effects of factors, covariates, and interactions were tested; *p*-values of post hoc multiple comparison tests were Sidak-adjusted; the level of significance was set to *p* < 0.05. All data were analyzed using the statistical software package IBM^®^ SPSS^®^ 29.0.

## 3. Results

A total of 11 spines, each with 22 vertebrae, were extracted from human body donors. Per vertebra, we obtained three cancellous bone cylinders. Finally, 726 micro-CT samples were compared with 726 sagittal sections on CT with regard to cancellous bone density. The 11 spines were derived from five male and six female donors, 66 to 91 years old (79.1 ± 7.5). The available information about medical histories is limited to the cause of death. A summary is provided in Table 1.

### 3.1. QCT

The mean overall bone mineral density of 11 spines, measured from lumbar vertebra L1 to L3, yielded a value of 58.7 ± 27.2 mg/cm^3^, which indicates severe OP. In general, a bone mineral density below 80 mg/cm^3^ is interpreted as osteoporosis [5]. At a bone mineral density below 60 mg/cm^3^, we found more fusion fractures in the thoracic and thoracolumbar regions (*p* = 0.012). No fractures were seen in the cervical spine.

#### Density of Cancellous Bone in Hounsfield Units on the CT Image

**Figure 3:**
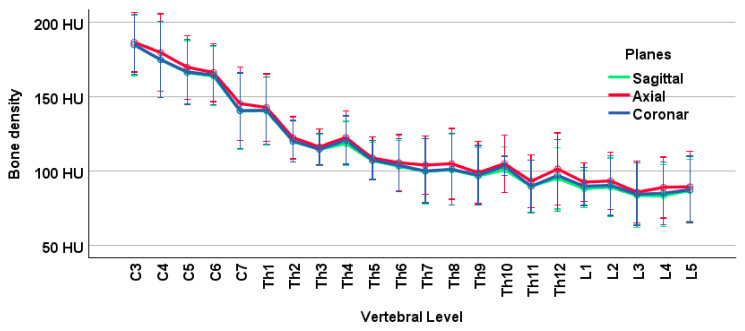
Cancellous bone density in Hounsfield units (HU) reduces along the spine from C3 to L5 (*p* < 0.001). Each vertebra was measured in 3 sectional planes (sagittal, axial, coronary). In all sections, we observed similar reducing values of bone density along the spine. Bone density is greater in the axial section than in the other two sectional planes. The average vertebral bone density (*n* = 11) of each sectional plane is shown as estimated marginal mean with the respective 95% confidence interval. Covariates appearing in the linear model are evaluated with age = 79.1 years and body mass index = 21.9 kg/m^2^.

Cancellous bone density (HU) fell from C3 downward (186 ± 25.9) to L5 (88.1 ± 29.1: *p* < 0.001) (Figure 3). Generally, the axial section (119 ± 17.2) yielded higher HU values than the sagittal (116 ± 16.8) or coronary sections (117 ± 16.4). Men (157 ± 22.3; *n* = 5) had higher bone densities than women (77.6 ± 18.9; *n* = 6; *p* = 0.002). All three sectional planes reflected the gender difference to the same degree (*p* = 0.003 each). Men had higher density values on the axial image than on the sagittal (*p* < 0.001) or coronary images (*p* = 0.007), but the latter two planes did not differ from each other (*p* = 0.256). In women, we found no significant density differences in the sectional planes (*p* ≥ 0.111).

On average, the donors had experienced 1.8 ± 1.1 fractures (Table 1). Vertebral fractures (VF) differed in numbers and showed the following differences in density: ≤1 fracture 146 ± 15.8; more than 1 fracture 89.1 ± 14.4 (*p* = 0.002). At a maximum number of one VF, there were differences between the axial and sagittal sections (*p* = 0.001), as well as the axial and coronary sections (*p* = 0.017). On the other hand, the comparison of HU values from the sagittal and coronary images revealed no significant difference (*p* = 0.236). In body donors with more than one VF, we also found significant differences in the comparison of HU values in the axial and sagittal sections (*p* < 0.001), as well as the axial and coronary sections (*p* = 0.024). Again, the comparison of sagittal and coronary sections revealed no significant difference (*p* = 0.060). Men with a maximum of one fracture had higher cancellous bone densities (206 ± 17.6) than men with more than one sustained fracture (109 ± 15.3; *p* = 0.003); the same was not observed in women (85.5 ± 19.0; 69.7 ± 13.8; *p* = 0.335). However, men with ≤1 fracture (*p* = 0.005) and more than 1 fracture (*p* = 0.014) had higher cancellous bone densities than women in the respective fracture groups.

### 3.2. Micro-CT Compared with CT

#### 3.2.1. Micro-CT and CT Scans Show Graphically Different Courses of Bone Density Values

**Figure 4:**
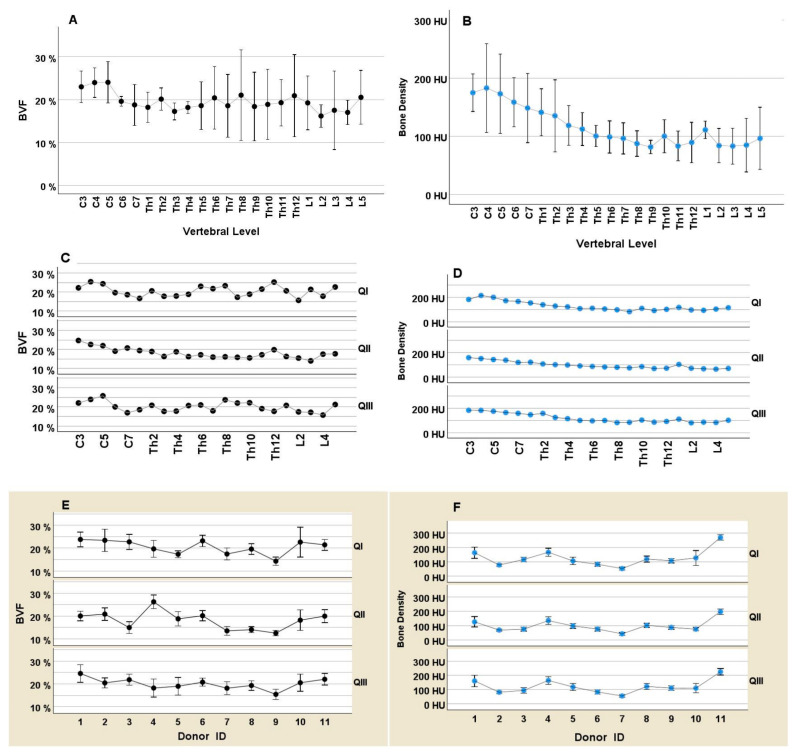
Graphic comparison of the two procedures for the determination of cancellous bone density with the aid of micro-CT (BVF%; left-sided panel, black dots) and CT (HU; right-sided panel, blue dots) with reference to the three quadrants QI, QII, QIII. **Above** (**A**) BVF% and (**B**) HU: Overall values for *n* = 11 body donors as profiles along the vertebrae C3-L5, averaged for 3 quadrants. A reduction in bone density along the spine is observed with both procedures but in different degrees. Data are given as means with their 95% confidence intervals. **Center** (**C**) BVF% and (**D**) HU: Individual views of the quadrants QI, QII, QIII. Each data point expresses the mean value for *n* = 11 body donors. The CT measurement yields, independent of the measured quadrant, uniformly reducing values. The micro-CT measurement shows different profiles in the quadrants, highlighting the importance of the measured quadrant. **Below** (**E**) BVF% and (**F**) HU: profiles of bone density for 11 donors in the investigated quadrants. In this regard as well, CT yields uniform profiles independent of the quadrant, but micro-CT does not. Data are given as means with their 95% confidence intervals.

A reduction in cancellous bone density from C3 to L5 was seen in CT investigations, as well as in the BVF values on micro-CT (C3: 25.0 ± 3.42; L5: 21.1 ± 7.56; *p* = 0.018). The values registered by the two procedures ran different courses (Figure 4). The differences were clearly seen on longitudinal sections over the 22 vertebrae in general (A vs. B), as well as divided into the 3 quadrants (C vs. D), and also in the 11 body donors (E vs. F).

#### 3.2.2. Gender and Fracture Status Influence Bone Density Measurements

Gender (*p* = 0.002) and the number of existing VF (≤1 vs. >1; *p* = 0.001) were independent factors influencing cancellous bone density, regardless of the procedure used. However, age (*p* = 0.227) and BMI (*p* = 0.091) had no effect.

#### 3.2.3. Micro-CT and CT Investigations Reveal Gender Differences with Varying Clarity

**Figure 5:**
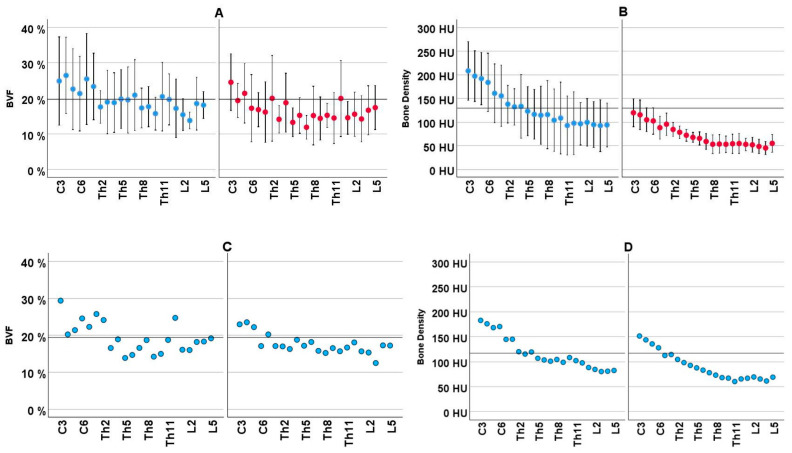
Bone density measurements (on the left side as bone volume fraction BVF%, on the right side in Hounsfield units HU) in relation to the two influencing factors: gender (**upper panel**) and the number of sustained vertebral fractures (VF), (**lower panel**), in relation to the location of the vertebral body. (**A**) BVF% of male (blue, *n* = 5, group mean = 19.7% as reference line) vs. female (red, *n* = 6). The mean density values in men serve as a reference line so that the below-average values in women become obvious. Data are shown as means with their 95% confidence intervals. (**B**) HU of male (blue, *n* = 5, group mean = 130 HU as reference line) vs. female (red, *n* = 6). (**C**) BVF% of the group with at most one experienced VF (**left**, *n* = 3, group mean = 19.4% as reference line) vs. the group with more than one VF (**right**, *n* = 8). Most of the shown vertebral BVF means of those with more than one fracture are smaller than the reference value of 19.4%. (**D**) HU of the group with at most one experienced VF (**left**, *n* = 3, group mean = 117 HU as reference line) vs. the group with more than one VF (**right**, *n* = 8). Data are given as means. Expected gender- and fracture-related differences in bone density are seen with both procedures. The higher bone density values on CT in HU were statistically confirmed in men as well as in the group with a maximum of one VF (*p* = 0.002 for each).

The two procedures (micro-CT and CT investigation) differed in their degrees of reflecting gender-related differences in cancellous bone density (Figure 5A vs. Figure 5B). Micro-CT investigation (A) revealed higher BVF values in men (*n* = 5) than in women (*n* = 6). On CT investigation (B), the gender difference was seen graphically on the fine scale in the individual vertebrae of the cervical, thoracic, and lumbar spine, and it was also statistically significant (*p* = 0.002).

#### 3.2.4. Gender Differences Based on HU Values Were Independent of the Investigated Quadrants

The CT investigation showed higher HU values in men than in women for each of the three quadrants: Q1 (*p* = 0.002), QII (*p* = 0.003), and QIII (*p* = 0.003). In contrast, the micro-CT investigation showed no significant gender difference in QI (*p* = 0.850), QII (*p* = 0.870) or QIII (*p* = 0.378). In women, both procedures revealed no differences between the three quadrants (*p* ≥ 0.061); the same was true of micro-CT measurements in men (*p* ≥ 0.672). Only CT measurements in men showed differences in the quadrants (*p* ≤ 0.011).

#### 3.2.5. CT and Micro-CT Investigations Differ in Their Depiction of Fracture Status

The CT measurements showed differences between the subgroups of ≤ 1 fracture (*n* = 3) and more than 1 fracture (*n* = 8; *p* = 0.002, Figure 5D). The micro-CT measurements (C) were 21.9 ± 3.03 and 18.5 ± 2.77 (*p* = 0.162). Micro-CT revealed no differences between fracture groups for women (*p* = 0.246) or for men (*p* = 0.431). The CT investigation, on the other hand, revealed higher HU values in men at a maximum number of one versus more than one sustained vertebral fracture (*p* = 0.003).

## 4. Discussion

The present investigation permitted a comparison of cancellous bone densities in all segments of the spine from 11 body donors aged 66 to 91 years, measured with two different procedures. Besides, for the first time a study yielded data from aspirations of different quadrants in a micro-CT investigation, and Hounsfield units from similar quadrants of a CT investigation. The BMI in the entire group is considered normal, BMI values below 22 are related to a rising fracture rate [13]. All investigated probands had bone mineral density values below 80 mg/cm^3^ on densitometry and therefore suffered from OP [14]; fusion fractures are obligatory at values below 60 mg/cm^3^ [8]. The age of the body donors contributed significantly to these findings [15,16,17]. A rise in fracture rates was seen especially beyond the age of 70 years [18]. Fractures were typically distributed in the thoracolumbar and lumbar portions of the axial skeleton [9]. One explanation for this fracture cascade along the spine could be the pre-existing curvatures of the spine [19]. The turning point of the curvature of thoracic kyphosis is in the mid-region of the thoracic spine (Th7, Th8). Due to the greater curvature, there are higher bending moments and compression loads at this site. This notion is supported by the fact that an increase in curvature—which is equivalent to greater kyphosis which develops in the course of life and is especially significant in osteoporosis—results in greater bending loads. Likewise, the elevated fracture risk at the junction of Th12 and L1 is possibly explained by greater mobility in the lumbar spine and consequently higher compression loads. Vertebral fractures of the thoracic and lumbar spine belong to the 10 most frequent fracture entities in Germany [3].

In the present investigation, L1 was affected most frequently by a fracture, independent of gender. It was interesting to note that L1 had a model-adjusted mean value of 90.2 HU and thus a higher cancellous bone density than L4 with 86.0 HU. Thus, it may be assumed that, in addition to cancellous bone density, the above-mentioned factors play an important role in the number of sustained fractures.

No fractures of the cervical spine were observed in the present study. Montemurro et al. [20] investigated the significance of a Y-shaped trabecular bone structure (TBS) in the odontoid process of the axis (C2 vertebra) in patients with cervical spine injuries. The researchers concluded that the Y-shaped TBS plays a crucial role in the biomechanical structural dynamics of the C1-C2 joint and has significant clinical relevance for dens fractures [20]. These results suggest that the absence of this specific bone structure may be associated with an increased risk of dens fractures in cervical spine injuries.

After analyzing their data from human vertebral bodies, Shin et al. [21] conclude that the superior region of the vertebral bodies shows a higher biomechanical susceptibility, which could explain its dominant role in osteoporotic vertebral fractures. The authors recommend paying particular attention to the use of bone mineral density measurements of the superior vertebral body region using lateral DXA (Dual-energy X-ray absorptiometry) when diagnosing osteoporosis. Lateral spine DXA offers several advantages over conventional posterior–anterior measurement. It allows for a more accurate assessment of trabecular bone density in the vertebral body by excluding posterior elements and degenerative changes. Studies have shown that lateral DXA correlates better with trabecular bone density measured by quantitative computed tomography [22,23]. Additionally, lateral DXA demonstrates a stronger association with age and shows a steeper decline in bone density with increasing age compared with anterior–posterior measurement [23]. Lateral DXA can detect osteopenia and osteoporosis more frequently than anterior–posterior measurement, especially in patients with degenerative changes in the spine [23,24]. This makes it a useful tool for early detection of bone mass loss. However, there are some limitations: the precision and accuracy of lateral measurement are slightly lower than anterior–posterior measurement [23], and there is a lack of established reference values and diagnostic criteria for lateral DXA. Therefore, lateral spine DXA is currently not routinely recommended for the diagnosis of osteoporosis or monitoring of bone mass loss. However, it can provide valuable information as a complementary measurement, particularly in patients with significant degenerative changes in the spine, where anterior–posterior measurement might overestimate bone density [24].

A study by Zhao [25] and colleagues examined the differences between cranial and caudal endplates of the spine. The researchers found that the upper (cranial) endplates were significantly thinner than the lower (caudal) ones. In midsagittal sections, the difference was approximately 14%, while in pedicular sections, a difference of about 11% was observed. In addition to thickness, the optical density of the trabecular bone immediately adjacent to the endplates was also investigated. Here, it was shown that the density in the cranial region was about 6% lower than in the caudal region. Based on these findings, the scientists concluded that the thickness of the endplate alone is not sufficient to explain the more frequent failure in the upper region of the vertebral body. Rather, the biomechanical properties of the endplate seem to be closely linked to the characteristics of the underlying trabecular bone. These results highlight the complexity of vertebral body structure and indicate that multiple factors must be considered when assessing the stability and load-bearing capacity of the spine.

Measurements of HU from nearly complete spines (C3-L5), including micro-CT investigations, have rarely been performed so far. HU shows a normalized index of attenuation of the X-ray beam, based on a scale from 1000 for air and 0 for water. The HU value of bone is typically between 300 and 3000 [26]. With the aid of HU, we can make statements about bone density. Schreiber et al. [26] determined a significant correlation between T-scores on bone densitometry and HU of the same vertebra.

Our data showed a reduction in bone density in HU from cervical vertebra C3 to lumbar vertebra L5. Schröder et al. [9] investigated 624 vertebrae from cervical vertebra C3 to sacral vertebra S2, extracted from 26 body donors by using a manually positioned region of interest in cancellous bone. The authors determined the mean value from axial, sagittal, and coronary sections. Their investigation revealed a similar course of HU values over the spine as those registered in the present investigation. The highest HU was noted in the cervical spine. They conclude that a loss of bone mineral density in vertebral cancellous bone leads to a higher risk of fractures, but a value lower than the fracture-critical threshold is not achieved in the cervical spine even in the presence of evident osteoporosis.

In contrast to Schröder et. al. [9], the present investigation compared HU values from the axial, sagittal, and coronary sections of the same vertebrae in order to detect differences, if any, in the various sectional planes. The highest HU values were noted in the axial sections. Marinova et al. [27] routinely evaluated chest and abdomen CT data from 234 patients aged on average 59 years and found no significant intra-individual differences between HU values in the axial, sagittal, and coronary planes. The authors assume that HU values are similar regardless of the sectional plane. In a further investigation performed by Kim et al. [28], the CT data of the lumbar spines of 100 patients aged over 50 years were evaluated. The authors also found no difference between the axial, sagittal, and coronary planes when screening for osteoporosis on CT, and concluded that all three planes could be used equally for the measurement of bone mineral density on CT. For the sectional planes, the authors compared the areas under the ROC curve (AUC) and the intra-class correlation coefficient (ICC). Kim et al. [28] achieved cut-off values of 110 HU in the coronary plane, 112 HU in the axial, and 112 HU in the sagittal planes.

In contrast, Zhang et al. [29] investigated 1338 patients aged on average 61.9 years and showed that measurements in the axial plane were on average 9 HU higher than those on sagittal measurements. The authors used the thoracic vertebra 7 for their measurements. With regard to the differentiation between osteoporosis and no osteoporosis, the sagittal plane showed greater diagnostic efficacy. For each one-unit reduction in the sagittal CT attenuation value, the risk of osteopenia or osteoporosis rose by 3.6 percent. The authors recommend a bone densitometry investigation for all patients with a sagittal CT attenuation value of <113.7 HU in the 7th thoracic vertebra.

The observation made in the present investigation that the HU values in the axial plane are higher than those in the sagittal and coronary planes can be explained by several radiological and anatomical factors. On the one hand, the partial volume effect [30] plays a decisive role in this phenomenon. In the axial plane, the region of interest (ROI) can be defined more precisely in cancellous bone, whereas sagittal and coronary reconstructions frequently include cortical bone or surrounding soft tissue. This results in the averaging of density values and, consequently, lower HU values in these planes. On the other hand, the trabecular structure of cancellous bone in the vertebrae is not isotropic. Due to the vertical arrangement of trabeculae, more bone substance per volume is registered in the axial plane, resulting in higher density values in this plane. Technical aspects of CT imaging, such as the selected slice thickness and reconstruction are also worthy of mention. The non-contrasted axial acquisition of CT data frequently yields a higher resolution than reconstructed sagittal and coronary images. Thinner sections in the axial plane may lead to more precise measurements and consequently higher HU values. The algorithms used for sagittal and coronary reconstructions may lead to a slight smoothing of data, which may influence the determined HU values. In order to minimize this discrepancy and obtain consistent values, the ROI should be established carefully in all three planes while avoiding cortical bone and marginal structures. Furthermore, if one wishes to obtain representative values, the formation of a mean value from the measurements of all three planes [9] appears to be meaningful only when there are no significant differences between the sectional planes. These conclusions underline the importance of a careful and consistent technique of measurement when determining HU values in vertebral bodies, especially in the context of bone density measurements and for the assessment of fracture risk.

Micro-CT investigations of the cancellous bone density of vertebrae from all segments of the spine are laborious and have been scarcely described so far. More recent studies [10,11] support our data showing that the bone volume fraction (BVF) reduces from the cervical to the lumbar spine.

In the present investigation, gender and fracture numbers, in contrast to age and BMI, were independent factors influencing cancellous bone density. In their study of 299 patients aged 65 to 90 years, Zou et al. [31] showed that, independent of age and gender, patients with a fragility fracture in the vertebrae had significantly lower HU values (on average 66 HU) than patients without a vertebral fracture (101.5 HU). The authors assume that HU values may serve as useful indicators, with high specificity (60 HU) and sensitivity (100 HU) for the identification of patients with a fracture risk [31]. In contrast to Zhou et al., our study was not limited to vertebrae in the thoracolumbar junction. Rather, we studied all segments of the spine.

In our study, the gender-specific differences in bone density values were significant and were shown in different degrees of clarity by the two methods. In the HU analysis, men had, as expected, significantly higher density values than women, especially in the axial section, which is indicative of differences in bone density and possibly also differences in bone quality and loads between genders. Women, on the other hand, had no significant differences in the sectional planes, which may be a sign of a more homogeneous distribution of bone structure. The micro-CT method revealed no significant gender differences in any of the quadrants, which is indicative of its limitations in clinical use.

Our results show that HU values on CT and BVF values on micro-CT investigation have different properties in terms of sensitivity in the determination of bone density. While HU values revealed uniform profiles of bone density over the vertebrae in the various quadrants and in individual body donors, BVF values were more heterogeneous in the quadrants. Particularly the bone density profile over the vertebral body in the central quadrant II showed the expected uniform reduction in values. The “noisy” data on micro-CT investigation may be an indication of the fact that this method of measurement is more strongly influenced by the choice of the quadrant. At the same time, the direction of aspiration used for the sampling procedure appears to play a role. Schröder et al. [12] compared ventrodorsal cancellous bone puncture with the craniocaudal one in 20 body donors aged on average 79.4 years. After completion of their study, the authors concluded that ventrodorsal puncture yields more subcortical structures, especially from the marginal areas of the vertebrae. Furthermore, on the Bland–Altman diagram, the authors showed the presence of a bias related to the selected direction of puncture in that a low BVF provides similar values, but a higher BVF yields markedly deviating values.

In contrast, the HU measurements on CT appear to be independent of the quadrants, which makes them more robust for clinical diagnostic investigation in order to obtain a comprehensive view of bone density.

Knowledge of HU for individual vertebral bodies offers significant advantages in the surgical treatment of osteoporotic fractures, enabling more precise preoperative risk assessment. Studies have demonstrated that low HU values (<120–150 HU) indicate reduced bone density and an increased risk of osteoporosis, allowing for better estimation of the risks of screw loosening, implant failure, or adjacent fractures [32,33]. This information can substantially influence operative planning, with consideration given to additional stabilization measures such as cement augmentation or extended instrumentation in cases of low HU values [32,34]. Compared with conservative management, HU-based surgical planning offers the advantage of improved patient selection. Patients with very low HU values may benefit more from conservative therapy due to increased surgical risks, while those with sufficient HU values may achieve better functional outcomes through surgical intervention [35]. Furthermore, preoperative HU measurement can provide insight into long-term prognosis, as low HU values are associated with an increased risk of adjacent fractures and poorer clinical outcomes [36]. In conclusion, knowledge of HU values enables more precise and individualized treatment planning for osteoporotic vertebral fractures. It can contribute to minimizing complication risks and improving long-term outcomes [37,38]. Compared with purely conservative therapy, this approach offers a more informed basis for decision-making regarding surgical versus non-surgical management and allows for better risk stratification [6,39]. The integration of HU measurements into clinical practice could thus lead to the optimization of treatment strategies for patients with osteoporotic vertebral fractures [40,41].

Comparison of HU determined on CT and BVF measured on micro-CT shows that HU is probably more suitable for the demonstration of gender differences, especially in the cervical, thoracic, and lumbar spine, whereas these differences are not seen clearly on micro-CT. These data confirm that the HU method might be more suitable for clinical applications in which the investigator wishes to perform a more detailed assessment of bone density in various anatomical regions. On the other hand, Parsa et al. [42] performed a correlation analysis between BVF and HU in 20 mandibles of human cadavers and noted an excellent correlation. Furthermore, given the high resolution and sensitivity of the micro-CT investigation with regard to microstructural properties of bone, it permits a more detailed analysis of trabecular microarchitecture, which makes it valuable for research applications. Chen et al. [43], as well as Gong et al. [44], report that the central and anterosuperior regions of the vertebra have a lower bone volume fraction (BVF) than the posterior region. The spatial distribution of bone density within vertebrae exhibits significant variations, as demonstrated by Banse and colleagues [45] in their study. Their findings suggest that the upper and anterior half of the vertebral body tends to have a lower density. These observations regarding regional variability in microstructure are of great importance for understanding gender- and age-related changes in bone substance within the spine. Consideration of structural aspects can contribute to a deeper understanding of various fracture patterns and risks [46]. Furthermore, microdamage to trabeculae plays a non-negligible role in assessing bone strength. These micro-injuries are partly related to a reduction in bone volume fraction (BVF) [47]. Microdamage in the microarchitecture of vertebral bodies in osteoporosis refers to small structural defects in the trabecular bone that accumulate with increasing age and progressive osteoporosis. These microdamages occur in the form of linear microcracks, diffuse damage, or trabecular microfractures [48]. Understanding these microdamages and their relationship to bone architecture is crucial for assessing fracture risk and developing strategies to prevent osteoporotic VF.

Future research should be focused on establishing standardized values for bone density in various anatomical regions and demographic groups and utilize this information to improve clinical practice and personalized medicine.

The present investigation was conducted to compare two radiological procedures. It was confined to a case number limited by the existing material. The values obtained with both procedures for the demonstration of cancellous bone density differed by a power of ten. The results of subgroup (*n* ≤ 6) comparisons may be considered exploratory, and graphic comparisons were used to elucidate the statements. We exclusively used body donors of advanced age; no statements can be made about the bone structure of younger body donors. We also had very little medical history data about the donors, especially about the type and duration of any drug treatment or physical treatment they may have received for osteoporosis.

## 5. Conclusions

The present investigation provides more extensive insights into the complex relationship between bone density, gender, fracture status, and methods of measurement than hitherto known. It underlines the significance of selecting the suitable imaging procedure and parameters such as sectional planes and quadrants in order to perform an accurate diagnosis and administer specific treatment for osteoporosis, as well as other associated bone diseases.

The presented investigation shows that CT, as well as micro-CT, may contribute to a better understanding of osteoporosis.

A major advantage of the CT investigation is its clinical applicability. HU measurements from routine CT scans can be used as a screening tool for osteoporosis, especially in patients who undergo CT investigations for other reasons. This would dispense with additional imaging procedures and radiation. In view of the limited availability of dual-energy X-ray absorptiometry, this makes CT scans an interesting tool for smaller clinics.

## Figures and Tables

**Table 1:** Medical history data of the entire group.

	Body Donors (*n* = 11)
Age (years)	79.1 ± 7.5
Gender (male/female)	5/6
Body mass index (kg/m^2^)	21.9 ± 5.5
Number of sustained fractures (≤1 fracture/>1 fracture) Extracted segments	3/8 C3-L5
Bone density in the lumbar vertebrae 1 to 3 (mg/cm^3^) *	58.7 ± 27.3
Number of vertebral body fractures	1.8 ± 1.1
Number of vertebral fractures in relation to gender: overall/male/female (*n*)	
Th5	1/0/1
Th6	1/1/0
Th7	3/2/1
Th8	3/2/1
Th9	2/1/1
Th10	1/0/1
Th12	2/2/0
L1	5/1/4
L2	2/0/2
Number of investigated vertebrae (*n*)	242
Number of investigated cancellous bone cylinders (*n*)	726
Hounsfield units in the sagittal plane in QI to Q III (*n*)	726
Comparison of sectional planes in Hounsfield units (axial, coronary, sagittal) (*n*)	726

Data shown as numbers (*n*) or mean ± standard deviation (SD); * measurements based on quantitative computed tomography (QCT).

## Data Availability

The data presented in this study are available upon request from the corresponding author.

## Abbreviations

Al	aluminum
AUC	area under the curve
BMD	bone mineral density (mg/cm^3^)
BMI	body mass index
BVF	bone volume fraction
cm	centimeter
CS	cervical spine
CT	computed tomography
C1-7	cervical vertebra 1-7
DXA	dual-energy X-ray absorptiometry
EMM	estimated marginal means
Fig.	figure
g/cm^3^	gram/cubic centimeter
GE	General Electric
HU	Hounsfield units
IBM	International Business Machines Corporation
ICC	intra-class correlation coefficient
kV	kilovolt
L1-5	lumbar vertebra 1-5
LS	lumbar spine
mg/cm^3^	milligram/cubic centimeter
mg/mL	milligram/milliliter
Micro-CT	micro-computed tomography
Mio.	millions
Ml	milliliter
Mm	millimeter
OP	osteoporosis
PMMA	polymethylmethacrylat
Q I	Quadrant I
Q II	Quadrant II
Q III	Quadrant III
QCT	quantitative computed tomography
ROC	receiver operating characteristic
ROI	region of interest
SD	standard deviation
TBS	trabecular bone structure
Th1-12	thoracic vertebra 1-12
TS	thoracic spine
VF	vertebral fractures
µm	micrometer
µA	microampere
°C	degrees Celsius
